# Spontaneous Coronary Dissection Induced Cardiac Arrest During Posterior Instrumentation in Prone Position: A Case Report

**DOI:** 10.7759/cureus.36527

**Published:** 2023-03-22

**Authors:** Özgecan P Zanbak Mutlu, Deniz Mutlu, Barkın Kültürsay

**Affiliations:** 1 Anesthesiology and Reanimation, Bahçelievler State Hospital, Istanbul, TUR; 2 Cardiology, Istanbul University-Cerrahpasa, Istanbul, TUR; 3 Cardiology, Kartal Kosuyolu Heart Education and Research Hospital, Istanbul, TUR

**Keywords:** perioperative myocardial infarction, intraoperative, ventricular fibrillation, prone, spontaneous coronary artery dissection, cardiac arrest

## Abstract

Intraoperative cardiac arrest (ICA) is a crucial property of morbidity and mortality for patients undergoing surgical operations. Spontaneous coronary artery dissection (SCAD) is an important cause of ICA and perioperative myocardial infarction, especially in young women. In this case report, we presented the successful management of SCAD-induced ICA in a 46-year-old female patient who underwent posterior spinal instrumentation in the prone position due to lumbar intervertebral disc extrusion.

## Introduction

In the contemporary surgical era, the number of operations is growing exponentially worldwide. On average, the complication rate associated with noncardiac surgery is reported to be 7-11%, and the mortality rate is 0.81-5% [1]. Intraoperative cardiac arrest (ICA) is a fundamental entity of morbidity and mortality for patients undergoing surgical operations. The data is heterogenous regarding the incidence of the ICA; recent studies showed the incidence rate was 5.6 per 10.000 procedures, the mortality rate was 58.4%, and increased with age, male gender, and American Society of Anesthesiologist's physical status [2].

Owing to this catastrophic event, ICA has received considerable scholarly attention in recent years. The incidence of ICA associated with noncardiac surgery has decreased evidently with the constantly evolving techniques and changing clinical practices [3]. However, anesthesiologists should be aware of the frequency, risk factors, and appropriate treatment techniques for this fatal condition. One of the possible causes of ICA in young patients is spontaneous coronary artery dissection (SCAD); due to the development of intravascular imaging, awareness of the diagnosis has increased [4,5]. We aim to present a case of SCAD-induced ICA during lumbar stabilization in the prone position.

## Case presentation

A 46-year-old female (height: 165 cm, weight: 70 kg, BMI: 25,7) had a history of hypertension and presented in the outpatient clinic due to steppage gait at the right foot and severe pain. Magnetic resonance imaging (MRI) demonstrated an intervertebral disk protrusion at L4-5 and an extrusion at L5-S1, respectively. Her only chronic medication was valsartan 160 mg. No abnormality was detected in the preoperative physical examination, electrocardiography, and laboratory values. The patient was intubated with 2 mg midazolam, 150 mcg of fentanyl, 120 mg propofol, and 50 mg rocuronium. Anesthesia was maintained with sevoflurane. After the induction of anesthesia, the patient was positioned in the prone position. The patient underwent minimally invasive transpedicular interbody fusion at L4-L5 and S1. The procedure duration was 86 minutes, and the patient remained stable from respiratory (SpO_2_: 98-100%, EtCO_2_: 30-35 mmHg) and hemodynamic (average mean arterial pressure (MAP): 82.51 mmHg) aspects throughout the surgery. Intraoperative infusion of 2200 ml of crystalloid was administered to the patient with total bleeding of 200 ml, and urine output was 300 ml. While wound closure had been performed, the patient developed cardiac arrest with the rhythm observed as the ventricular fibrillation (VF) on the monitor. Due to the open surgical wound, the patient was urgently rotated from the prone position to the lateral, and anteroposterior defibrillation succeeded with biphasic 220 joules. After the return of spontaneous circulation, the wound was quickly closed in the lateral position and turned to the supine position. Due to labile and hypotensive blood pressure (BP) (70/44 mmHg), noradrenaline infusion started and was titrated according to invasive BP monitoring. Electrolyte disturbance, anemia (Hgb:10.8 g/dl), and respiratory and metabolic pathologies were excluded with arterial blood gas, biochemical and hematological studies. In addition, there was no hypo-hyperthermia detected. The patient was urgently transferred to the intensive care unit for further evaluation.

**Figure 1:**
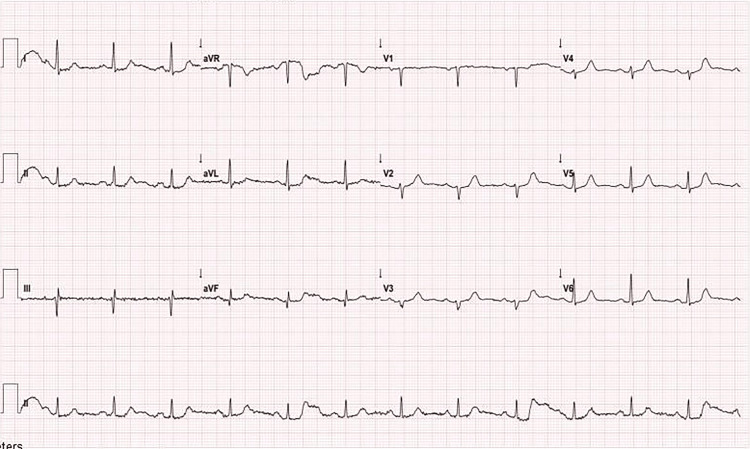
Post-cardiac arrest electrocardiography demonstrated sinus rhythm and lateral subtle ST segment depression

**Figure 2:**
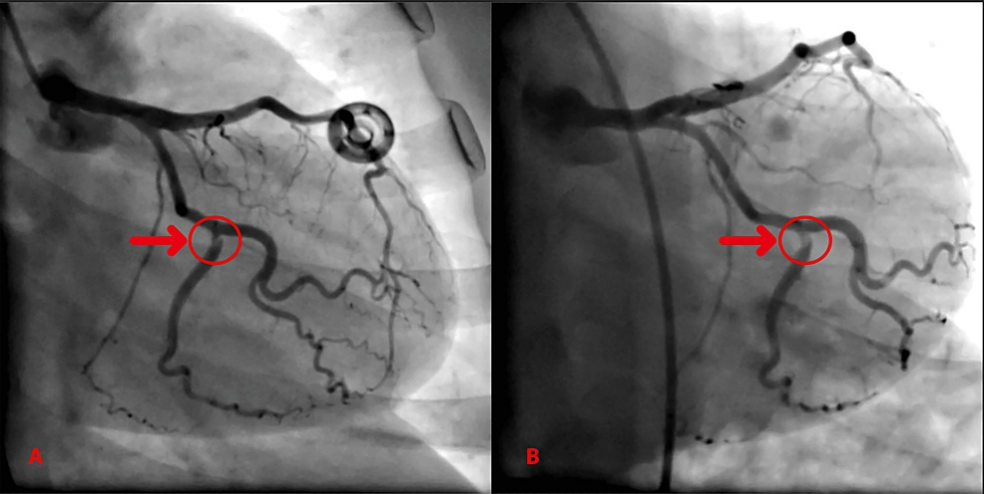
Coronary angiography during perioperative myocardial infarction A: Caudal position, arrow showed SCAD region in LCX, B: Left caudal position, arrow showed SCAD region in LCX. SCAD - spontaneous coronary artery dissection, LCX - left circumflex coronary artery

In the ECG, sinus rhythm and lateral subtle ST segment depression were detected (PR: 180 ms, HR:75/min, QTc:402ms) (Figure 1). Transthoracic echocardiography (TTE) demonstrated a mild to moderate mitral regurgitation and ejection fraction of 45% with an anterolateral left ventricular wall motion abnormality. Pericardial tamponade was excluded. The patient with a preoperative high specific cardiac Troponin T value (cTnT) of <5 ng/L was diagnosed with non-ST segment elevation myocardial infarction (NSTEMI) after a 90-fold increase in postoperative cTnT (471 ng/L). Subsequently, the patient was transferred to the cardiac catheterization laboratory (CCL), and her coronary angiogram (CAG) demonstrated nonobstructive SCAD in the proximal circumflex artery (Figure 2, Video 1).

**Video 1:** Coronary angiography during perioperative myocardial infarction shows SCAD on LCX SCAD - spontaneous coronary artery dissection, LCX - left circumflex coronary artery

Due to the nonobstructive nature and distal Thrombolysis in Myocardial Infarction (TIMI) III coronary flow, it was decided to follow up conservatively. The patient was extubated after hemodynamic stabilization was achieved. She was conscious and cooperative, and no signs were observed in her neurological examination. Bisoprolol 5 mg and acetylsalicylic acid (ASA) 100 mg were started. As a result of the high risk of bleeding due to the spinal operation, a P2Y12 inhibitor could not be added to the treatment. At week one, control CAG was normal. At the sixth-month follow-up, there was no complaint.

## Discussion

ICA is unique and differs from cardiac arrest in non-operative settings due to its witnessed nature, and underlying reasons can be frequently determined [6]. Among the ascribable reasons, vagal responses due to surgical manipulation, vagotonic agents, neuraxial blockade, inhalational and intravenous anesthetic overdose, malignant hyperthermia, hypoxemia, bronchospasm, acute coronary syndrome, cardiogenic and hypovolemic shock, pulmonary embolism, anaphylaxis, electrolyte disturbances, and tension pneumothorax can be counted [6]. Previous research has established that the incidence of ICA in noncardiac surgery detected approximately 5.6 per 100 000 cases [7]. Ostensibly it can be considered rare; knowing the proper care is critical for lowering the mortality risk. Management of the ICA relies on the fundamental reason for the event.

The prone position is linked with several problems due to pressure on anterior structures, causing hemodynamic alterations and difficulty in airway management owing to tracheal compression. ICA in the prone position is challenging, especially in spine surgery. The generalizability of published research on intraoperative-prone cardiopulmonary resuscitation (CPR) is problematic. Staartjes et al. [8] suggested not attempting CPR in the prone position and urgently preparing the patient for rotation after performing tamponade with surgical gauze or closing the wound with rescue sutures through all layers. Since the wound site was open and the defibrillator was easily accessible, anteroposterior defibrillation was performed urgently by positioning our patient in the lateral decubitus position. After one shock, return of spontaneous circulation (ROSC) was maintained, and we did not need to perform CPR. It is also consistent with the guideline recommendation that immediate cardioversion should be performed to witness tachycardia causes hemodynamic instability [6]. Recently, Cheskes et al. [9] demonstrated that anteroposterior defibrillation is superior to the standard method in providing sinus rhythm. Therefore, it can be argued that the method we used in our case can be suitable in cases where emergency cardioversion and defibrillation are needed for operations in the prone position.

Perioperative myocardial infarction (PMI) refers to acute myocyte injury with or without accompanying symptoms, ECG, or imaging evidence [1]. Systematic work-up and treatment of PMI are crucial. Firstly, 12-lead ECG, symptoms, and hemoglobin levels should be interrogated. If there is any ST segment abnormality or typical chest pain, severe anemia (Hgb< 8 g/dl) should be excluded. Subsequently, TTE and cardiac high-specific troponin T should be performed to overcome differential diagnosis. After the PMI diagnosis, it is recommended to load statin and aspirin based on the patient's bleeding status and refer the patient to the catheter lab for emergency CAG [1].

SCAD is a non-atherosclerotic, non-traumatic, acute coronary event characterized by spontaneous hematoma development in the tunica media of a coronary artery leading to coronary compression and myocardial ischemia [5]. Two hypotheses have been proposed to explain pathophysiology. The 'inside-out' hypothesis proposed that luminal blood flow directed to the tunica media due to endothelial disruption causes hematoma formation, whereas the 'outside-in' hypothesis proposed that hematoma is induced by adventitial vaso vasorum rupture [5]. The left anterior descending coronary artery is the most often affected coronary artery, accounting for 50% of all cases [10]. According to angiography, SCAD is classified into three types. Type 1 has multiple radiolucent lumens, type 2 has widespread stenosis with an abrupt change in artery caliber, and type 3 has localized or tubular stenosis that mimics atherosclerosis [11].

SCAD is more common in middle-aged women who have a lower cardiovascular risk [5]. Previous research has established that SCAD is the primary reason for approximately 35% of all ACS cases in women under the age of 50 [12]. In addition, it has been reported that approximately 70% of SCAD occurs in women [5]. Hypertension, familial history, connective tissue disorders, fibromuscular dysplasia, migraine, cocaine abuse, chemo-, and radiotherapy are all risk factors [5]. Also, pregnancy has been associated with SCAD. Women who are older at the time of their first childbirth and who are multigravida are at a higher risk of SCAD. Contraception and postmenopausal hormone usage, on the other hand, did not appear to raise the risk. There is no data on the relationship between absolute estrogen and progesterone levels and SCAD risk [5]. Among the precipitating factors, the common ones are extreme physical and emotional stress, valsalva, and retching [5]. As a result, clinicians should be aware of the risk factors prior to anesthesia and take precautions.

The primary aim of treatment is to restore or preserve myocardial perfusion and cardiac function [5]. Management of acute SCAD is examined under three headings: (1) clinically stable and no high-risk anatomy; conservative therapy with beta-blockers, single antiplatelet agent, and cardiovascular risk modification is recommended; dual antiplatelet therapy is contentious in conservative management due to the increased risk of intramural hematoma and true lumen compression, (2) clinically stable and left-main or severe proximal two-vessel SCAD; preferably coronary artery bypass graft (CABG) is recommended, (3) active/ongoing ischemia or hemodynamic instability; percutaneous coronary intervention (if the anatomy is feasible) or urgent CABG should be considered [5,11]. According to relevant guidelines, conservative therapy was chosen since our patient's lesion did not have high-risk anatomy and was nonobstructive; she was clinically stable in the cardiac catheterization laboratory [5,11].

To date, no randomized trial or reported case has assessed the impact of noncardiac operative stress on SCAD-induced PMI. However, there are several case reports of pregnancy. Newell et al. [4] reported it during an elective cesarean section under spinal anesthesia in a 37-year-old woman, successfully treated with the conservative approach. Our case differed for several reasons from the previously reported cases. Firstly, SCAD occurred during the prone position in the spinal operation. The literature on ICA during supine surgery in the prone position has highlighted the etiology of most cases related to air embolism [8]. The risk of air embolism was excluded for several reasons. Our patient experienced ICA after the operation's completion; she was monitored and appropriately positioned throughout the surgery. Furthermore, unlike the patients who experienced air embolism, our patient experienced VF instead of asystole. Secondly, there was no defined cardiac risk factor except hypertension and being a young female. Furthermore, our patient initially developed the VF rhythm; this also increased the ischemic suspicion.

## Conclusions

This article emphasizes that SCAD is an important cause of ICA and PMI, especially in young women, and can be solely triggered by surgical stress. Furthermore, points to be considered in diagnosis and treatment are highlighted, and it is vital to increase the awareness of medical doctors on this paramount pathology. Further research using controlled trials is needed to overcome this puzzling phenomenon.

## References

[1] Halvorsen S, Mehilli J, Cassese S (2022). 2022 ESC Guidelines on cardiovascular assessment and management of patients undergoing non-cardiac surgery. Eur Heart J.

[2] Nunnally ME, O'Connor MF, Kordylewski H, Westlake B, Dutton RP (2015). The incidence and risk factors for perioperative cardiac arrest observed in the national anesthesia clinical outcomes registry. Anesth Analg.

[3] Goswami S, Brady JE, Jordan DA, Li G (2012). Intraoperative cardiac arrests in adults undergoing noncardiac surgery: incidence, risk factors, and survival outcome. Anesthesiology.

[4] Newell CP, Seller C, Vizhi M, Turner N (2011). Case report: spontaneous coronary artery dissection during elective caesarean section under spinal anaesthesia. Anaesthesia.

[5] Hayes SN, Tweet MS, Adlam D, Kim ES, Gulati R, Price JE, Rose CH (2020). Spontaneous coronary artery dissection: JACC state-of-the-art review. J Am Coll Cardiol.

[6] Moitra VK, Einav S, Thies KC (2018). Cardiac arrest in the operating room: resuscitation and management for the anesthesiologist: part 1. Anesth Analg.

[7] Hinkelbein J, Andres J, Thies KC, DE Robertis E (2017). Perioperative cardiac arrest in the operating room environment: a review of the literature. Minerva Anestesiol.

[8] Staartjes VE, Schillevoort SA, Blum PG, van Tintelen JP, Kok WE, Schröder ML (2018). Cardiac arrest during spine surgery in the prone position: case report and review of the literature. World Neurosurg.

[9] Cheskes S, Verbeek PR, Drennan IR (2022). Defibrillation strategies for refractory ventricular fibrillation. N Engl J Med.

[10] Lettieri C, Zavalloni D, Rossini R (2015). Management and long-term prognosis of spontaneous coronary artery dissection. Am J Cardiol.

[11] Collet JP, Thiele H, Barbato E (2021). 2020 ESC Guidelines for the management of acute coronary syndromes in patients presenting without persistent ST-segment elevation. Eur Heart J.

[12] Nakashima T, Noguchi T, Haruta S (2016). Prognostic impact of spontaneous coronary artery dissection in young female patients with acute myocardial infarction: a report from the Angina Pectoris-Myocardial Infarction Multicenter Investigators in Japan. Int J Cardiol.

